# Response and Remission Rates Following High-Frequency vs. Low-Frequency Repetitive Transcranial Magnetic Stimulation (rTMS) Over Right DLPFC for Treating Major Depressive Disorder (MDD): A Meta-Analysis of Randomized, Double-Blind Trials

**DOI:** 10.3389/fpsyt.2018.00413

**Published:** 2018-09-07

**Authors:** Xu Cao, Chunshan Deng, Xiaolin Su, Yi Guo

**Affiliations:** ^1^Department of Neurology, Shenzhen People's Hospital, Second Clinical College, Jinan University, Shenzhen, China; ^2^Department of Neurology, Shenzhen University General Hospital, Shenzhen, China

**Keywords:** transcranial magnetic stimulation, major depression disorder, dorsal-lateral prefrontal cortex, meta-analysis, treatment

## Abstract

**Background:** High-frequency (HF) repetitive transcranial magnetic stimulation (rTMS) over the left dorsolateral prefrontal cortex (L-DLPFC) is the most widely applied treatment protocol for major depressive disorder (MDD), while low-frequency (LF) rTMS over the right DLPFC (R-DLPFC) also exhibits similar, if not better, efficacy for MDD. Therefore, a meta-analysis is warranted to compare the efficacy of the two protocols for MDD.

**Method:** We searched the literature from 1990 through to August 1, 2017 using MEDLINE, and the literature from 1995 through to August 1, 2017 using EMBASE, PsycINFO, the Cochrane Central Register of Controlled Trials (CENTRAL), SCOPUS, and ProQuest Dissertations and Theses (PQDT). We included randomized controlled trials (RCT) comparing the efficacy of HF rTMS over the L-DLPFC and LF rTMS over the R-DLPFC for MDD, which used response and/or remission rates as the primary endpoints, with and without sham-controlled.

**Results:** (1) The meta-analysis of the response rates was based on 12 studies, including 361 patients with MDD (175 for HF (> 5 Hz) over the L-DLPFC, and 186 for LF (<5 Hz) over the R-DLPFC; odds ratio = 1.08; 95%, confidence interval = 0.88–1.34). (2) The meta-analysis of the remission rate was based on 5 studies, including 131 MDD patients (64 for HF over the L-DLPFC and 67 for LF over the R-DLPFC; odds ratio = 1.29; 95% confidence interval = 0.54–3.10).

**Conclusion:** Both HF rTMS over the L-DLPFC and LF over the R-DLPFC demonstrated similar therapeutic efficacy for the treatment of patients with MDD. The results suggested that further investigation on treatment efficacy indicators before/during treatment is necessary and helpful for optimizing a personalized protocol for patients.

## Introduction

Transcranial magnetic stimulation (TMS) is a non-intrusive neuromodulation technique used to induce brief magnetic pulses of up to several Tesla in strength, via rapid discharging current of several thousand amperes through a stimulation coil (1, 2). The magnetic field can induce an electrical field in the cortex to depolarize superficial axons and to activate the neural networks when the coil is placed on a human head. The physiological effects induced by the electrical field depend on many TMS parameters, such as coil type and orientation, magnetic pulse waveform, stimulation frequency, and pattern. The consensus appeared to consider that high-frequency (HF, ≥ 5 Hz) stimulation could induce excitatory plasticity and low-frequency (LF, ≤ 1 Hz) stimulation could induce inhibitory plasticity in the cortex, based on motor evoked potential size changes in response to M1 stimulation in healthy subjects (3). Although this dichotomy is not entirely satisfying, many studies and clinical treatment protocols have been designed based on this principle.

Major depressive disorder is characterized by metabolic and neuronal activity asymmetry in the two prefrontal areas, showing elevated glucose and oxygen consumption as well as EEG activity on the right side, while it was suppressed on the left side, and the neural activity asymmetry correlated with clinical scores (4–7). Thus two main rTMS research protocols for the treatment of depression have been developed: LF stimulation on the right dorsolateral prefrontal cortex (R-DLPFC) (inducing inhibitory plasticity on the presumably hyperactive area), HF stimulation on the left DLPFC (L-DLPFC) (inducing excitatory plasticity on the presumably hypoactive area), or a combination of the two (8–10). A meta-analysis based on 29 randomized, double-blind, sham-controlled trials (RCTs), and 1,371 subjects with MDD showed that HF-rTMS was significantly effective in improving clinical scores compared to sham stimulation (11), and another meta-analysis consisting of eight RCTs and 263 subjects with MDD showed similar therapeutic efficacy of LF-rTMS for patients with MDD compared to sham stimulation (12). It is not clear which protocol is more effective or if they are equivalent for the treatment of MDD.

Here, we summarized the best available evidence to compare the therapeutic efficacy of the two most widely used protocols, LF-rTMS over the right DLPFC and HF-rTMS over the left DLPFC, for the treatment of patients with MDD. We performed a systematic review and meta-analysis of RCTs which directly compared the efficacy of the two protocols to compare the effects of the two protocols. We assessed both the response and remission rates.

## Materials and methods

### Search criteria

We identified the articles included in this meta-analysis according to the following criteria:

1. Searching MEDLINE from 1990 until August 1, 2017
2. Searching EMBASE, PsycINFO, the Cochrane Central Register of Controlled Trials (CENTRAL), SCOPUS and ProQuest Dissertations & Theses (PQDT) from 1 January 1995 until August 1, 2017
3. Screening the bibliography of the previous meta-analyses and reviews on rTMS for MDD.

**Figure 1:**
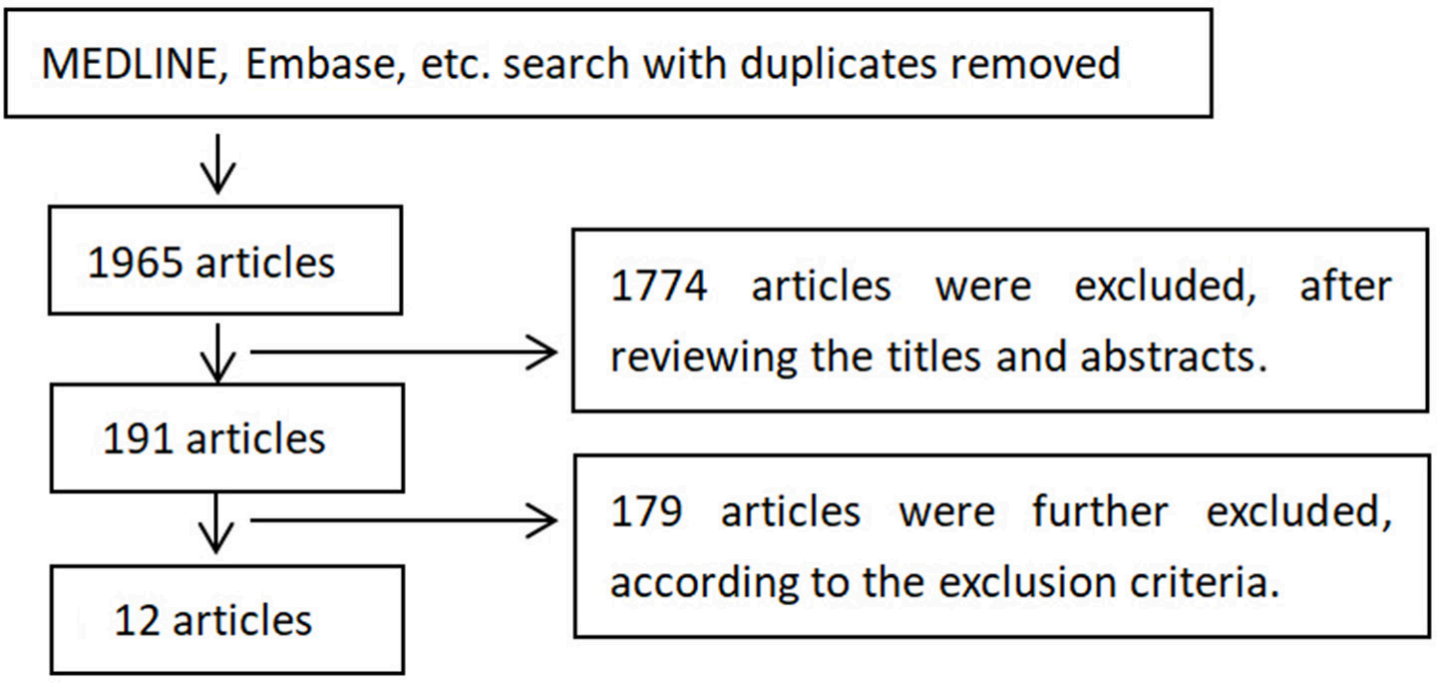
Selection of studies for inclusion. The 1,774 articles were excluded since most of them were other not research articles or not relevant to TMS treatment on depression. The 179 articles were excluded based on exclusion and inclusion criteria, including reviews and case reports, too few treatment sessions (<10 sessions), treatment protocols (compared rTMS with other treatments like transcranial direct current stimulation, non-random allocation, theta-burst stimulations), narrow diagnoses of depression, not reporting treatment efficacy, or rTMS not first time introduced to patients.

The key words “depression” and “transcranial magnetic stimulation” were used for searching above. The workflow to search and exclude studies is illustrated in Figure 1.

### In order to pool high quality and homogenous RCTS in our meta-analysis, we applied inclusion and exclusion criteria

#### Patients characteristics

Patients aged 18–80 years, diagnosed with primary major depressive episode (unipolar or bipolar) according to the Diagnostic and Statistical Manual of Mental Disorders (APA, 1994) or the International Classification of Diseases (WHO, 1992) criteria.

#### Treatment characteristics

LF-rTMS (≤5 Hz) over the R-DLPFC or HF-rTMS (≥5 Hz) over the L-DLPFC, were administered for ≥10 sessions, either as a monotherapy or as an augmentation strategy for patients with MDD.

#### Publication-related

We only included articles written in English.

#### Exclusion criteria

1. Studies that included patients with “narrow” diagnoses (e.g., postpartum depression, premenstrual dysphoric disorder, involutional depression) or secondary MDD (e.g., vascular depression, substance/medication-induced depression, psychotic depression)
2. Started rTMS treatment as a new antidepressant was introduced
3. Studies that did not report treatment efficacy (e.g., response or remission rate)
4. Studies that used non-randomized patient allocation.

The first two authors independently searched and identified studies to be included. The corresponding author decided whether the study should be included or excluded if there was disagreement between the first two authors.

### Procedure

#### Meta-analysis statistics

The response rate was used as the primary measurement of treatment efficacy. The response was defined as at least a 50% reduction in clinical evaluation scores (such as the Hamilton Depression Rating Scale, Montgomery Åsberg Depression Rating Scale, or Beck's Depression Inventory). The remission rate was used as the secondary measurement of efficacy, since not all studies reported these data.

RevMan 5.0 (Cochrane Information Management System) was used to perform statistical analysis. We used a Mantel–Haenszel fixed-effects model to calculate the combined ORs for each outcome and the chi-squared-based *Q*-test and I-squared index to assess heterogeneity. All tests were two-sided with statistical significance set to a *P*-value of 0.05 unless otherwise stated.

## Results

### Included studies

12 RCTs and 361 patients with MDD were included in the present meta-analysis (13–23). Among them, 175 patients were randomized to receive HF-rTMS over the L-DLPFC (mean age = 47.7 years, *SD* = 12.0 years, women = 57.1%), and 186 were randomized to receive LF-rTMS over the R-DLPFC (mean age = 49.6 years, SD = 10.9 years, women = 58.1%) (Table 1).

**Table 1:** Demographic and clinical characteristics of studies included in the meta-analysis.

**Study**	**N (HF vs. LF)**	**Female/male**	**Mean age, years (*SD*)**	**Primary diagnosis**	**TRD**
Hoppner et al. (19)	10 vs. 10	7/3 vs. 8/2	59.5 (6.8) vs. 52.0 (11.7)	All MDD	Yes
Fitzgerald et al. (16)	20 vs. 20	8/12 vs. 7/13	42.2 (9.8) vs. 45.6 (11.5)	19 MDD 1 BD vs. 19 MDD 1 BD	Yes
Chistyakov et al. (13)	10 vs. 12	5/5 vs. 9/3	59.3 (19.8) vs. 61.6 (8.7)	All MDD	Yes
Isenberg et al. (20)	14 vs. 14	8/6 vs. 8/6	43.4 (9.7) vs. 55.6 (9.7)	25 MDD 3 BD	Yes
Fitzgerald et al. (18)	15 vs. 11	8/7 vs. 5/6	42.4 (11.2) vs. 39.6 (10.0)	All MDD	Yes
Stern et al. (22)	10 vs. 10	6/4 vs. 7/3	53.2 (12.0) vs. 52.8 (9.5)	All MDD	Yes
Fitzgerald, et al., (17)	16 vs. 11	8/7 vs. 3/8	42.1 (9.3) vs. 46.5 (11.4)	All MDD	Yes
Rossini et al. (21)	32 vs. 42	23/9 vs. 30/12	53.4 (12.0) vs. 54.5 (11.9)	13 MDD 19 BD vs. 21 MDD 21 BD	Yes
Eche et al. (15)	6 vs. 8	2/4 vs. 6/2	50.8 (9.4) vs. 46.1 (16.3)	All MDD	Yes
Triggs et al. (23)	18 vs. 16	14/4 vs. 9/7	46.7 (15.3) vs. 48.5 (10.8)	18 MDD vs. 14 MDD 2 BD	Yes
Dell'Osso et al. (14)	13 vs. 20	5/8 vs. 11/9	52.1 (14.1) vs. 50.2 (8.5)	8 MDD 12 BD vs. 6 MDD 7 BD	10/13 vs. 15/20
Hu et al. (24)	12 vs. 13	6/5 vs. 5/7	27.4 (14.3) vs. 28.3 (10.3)	All BD	Yes

*HF, High-Frequency; LF, Low-Frequency*.

The mean number of rTMS treatment sessions was 14.6 (*SD* = 5.0), and the mean total rTMS pulses number for HF-rTMS was 19,708 (*SD* = 12,163), the mean total rTMS pulses number for LF-rTMS was 9,425 (*SD* = 7,621) (Table 2).

**Table 2:** rTMS treatment protocol characteristics of studies included in the meta-analysis.

**Study**	**rTMS Frequency (HF vs. LF)**	**Intensity (% rMT)**	**Sessions (n)**	**Total pulses(per session)**	**Country took place**	**Coil type**	**DLPFC**
Hoppner et al. (19)	20 vs. 1	90 vs. 110	10	800 vs. 120	Germany	8-shaped	5 cm
Fitzgerald et al. (16)	10 vs. 1	100 vs. 100	10	1,000 vs. 300	Australia	8-shaped	5 cm
Chistyakov et al. (13)	10 vs. 3	100 vs. 110	10	500 vs. 450	Israel	Circular	6 cm
Isenberg et al. (20)	20 vs. 1	80 vs. 110	20	2,000 vs. 120	USA	Not specified	5 cm
Fitzgerald et al. (18)	10 vs. 1	100 vs. 110	15	1,500 vs. 720	Australia	8-shaped	Not specified
Stern et al. (22)	10 vs. 1	110 vs. 110	10	1,600 vs. 1,600	USA	8-shaped	5 cm
Fitzgerald, et al. (17)	10 vs. 1	100 vs. 110	20	1,500 vs. 720	Australia	8-shaped	5 cm
Rossini et al. (21)	15 vs. 1	100 vs. 100	10	600 vs. 600	Italy	8-shaped	5 cm
Eche et al. (15)	10 vs. 1	100 vs. 100	20	2,000 vs. 120	France	8-shaped	5 cm
Triggs et al. (23)	5 vs. 5	100 vs. 100	10	2,000 vs. 2,000	USA	8-shaped	5 cm
Dell'Osso et al. (14)	10 vs. 1	80 vs. 110	20	750 vs. 420	Italy	Not specified	5 cm
Hu et al. (24)	10 vs. 1	80 vs. 80	20	1,200 vs. 1,200	China	8-shaped	5.5 cm

*HF, High-Frequency; LF, Low-Frequency*.

### Response rate

**Figure 2:**
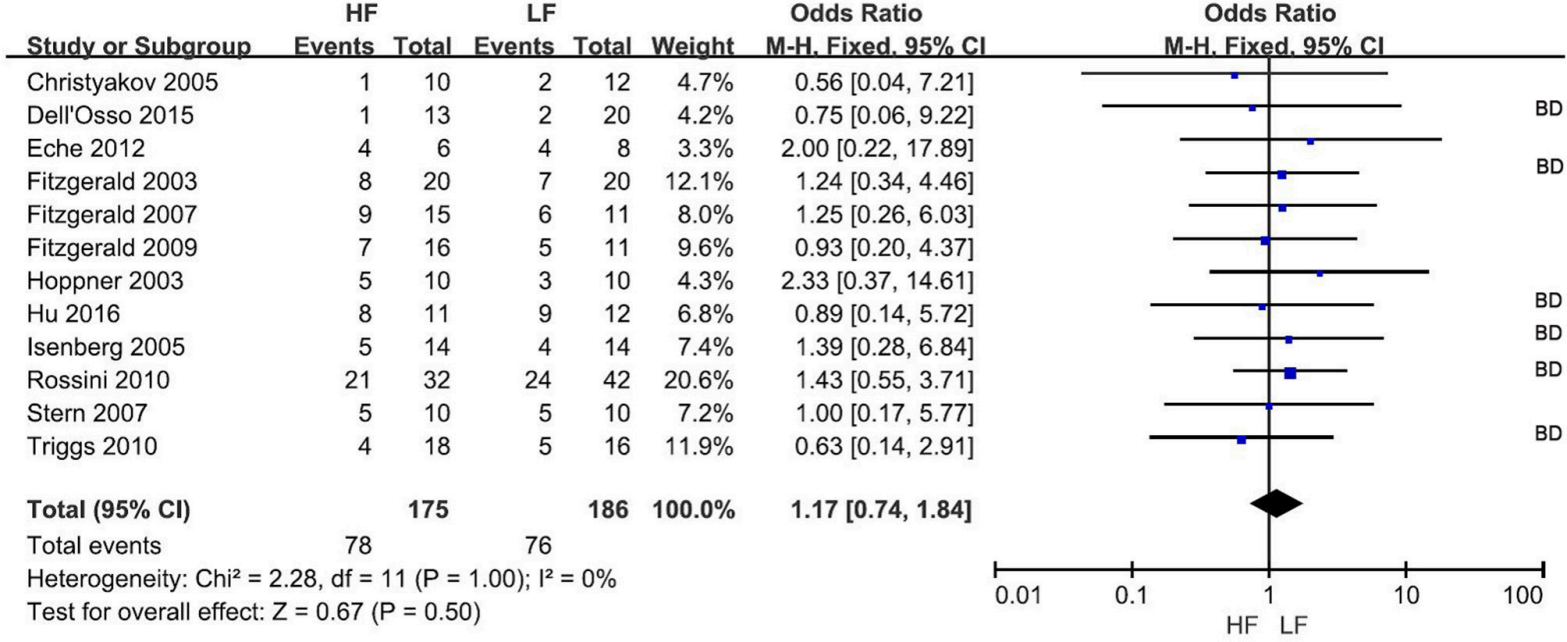
Meta-analysis of HF vs. LF-rTMS for MDD: response rate. HF, High Frequency; LF, Low Frequency; BD, Study in which more than one bipolar depressive patient involved.

The response rate was reported in all 12 RCTs (Figure 2) at the end of treatment. Overall, 78 of 175 subjects (44.6%) and 76 of 186 subjects (40.9%) receiving HF-rTMS over the L-DLPFC and LF-rTMS over the R-DLPFC, respectively, were classified as responders. The pooled OR was 1.08 (95% CI: 0.88–1.34, *Z* = 0.67, *P* = 0.50), indicating a comparative therapeutic efficacy between the two rTMS treatment protocols.

### Remission rate

**Figure 3:**
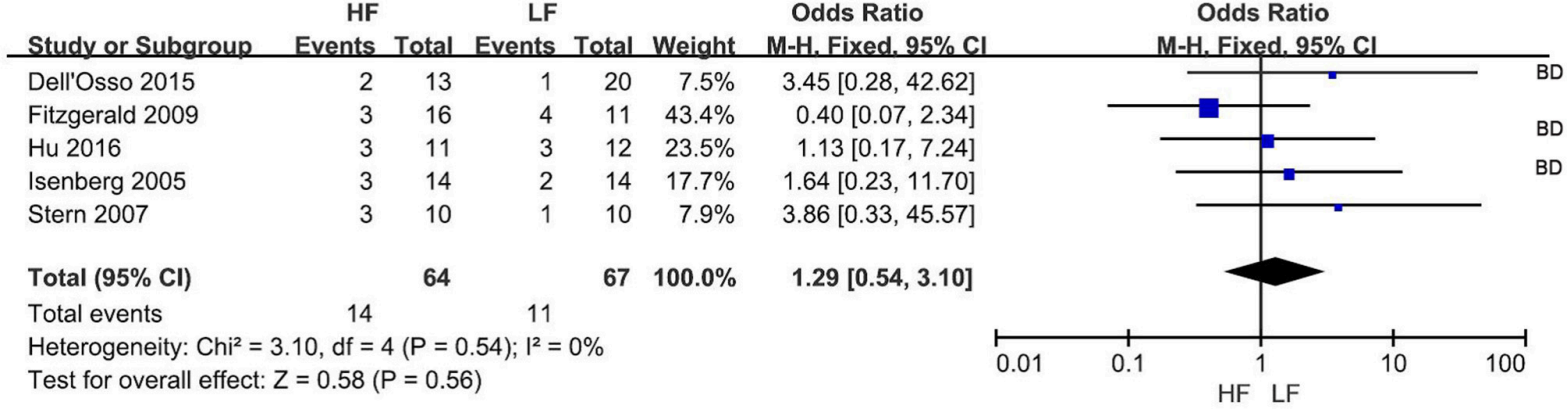
Meta-analysis of HF vs. LF-rTMS for MDD: remission rate. HF, High Frequency; LF, Low Frequency; BD, Study in which more than one bipolar depressive patient involved.

The remission rate was reported in 5 RCTs (Figure 3) at the end of treatment. Overall, 14 of 64 subjects (21.9%) and 11 of 67 subjects (16.4%) receiving HF-rTMS over L-DLPFC and LF-rTMS over R-DLPFC, respectively, were classified as remitters. The pooled OR was 1.29 (95% CI: 0.54-3.10, *Z* = 0.58, *P* = 0.56), indicating a similar therapeutic efficacy between the two rTMS treatment protocols.

### Dropout rate

Only 2 RCTs reported a dropout rate. Overall, 2 of 36 (5.5%) patients withdrew in HF-rTMS over the L-DLPFC group, and no patients in the LF-rTMS over the R-DLPFC group.

## Discussion

This is the first meta-analysis to compare the therapeutic efficacy of HF-rTMS over the L-DLPFC and LF-rTMS over the R-DLPFC for MDD in terms of both response and remission rates. The results showed that the two treatment protocols exhibited similar clinical efficacy (with 44.6 and 40.9% response rate for HF- and LF-rTMS, respectively; with 21.9 and 16.4% remission rate for HF- and LF-rTMS, respectively; with odds ratios of 1.08 and 1.29 for the response and remission rates, respectively). These results are consistent with those of a previous meta-analysis including a smaller number of RCTs and patients (25), and further extended the conclusion to include the remission rate. For the acceptability of different protocols, a meta-analysis including eight RCTs and 263 subjects showed that the dropout rate of the LF-rTMS treatment group was 5.3% (7/132) (12), while a meta-analysis including 29 RCTs and 1,371 subjects showed that the dropout rate of the HF-rTMS treatment group was 7.5% (55/730) (11). Moreover, considering safety issues, such as seizure induction, LF-rTMS over the L-DLPFC appears to be safer than HF-rTMS (26). To summarize, both HF-rTMS over the L-DLPFC and LF-rTMS over the R-DLPFC showed equivalent clinical efficacy for the treatment of patients with MDD.

Although both HF- and LF-rTMS over frontal areas have been proven effective for the treatment of MDD, it is challenging to conclude which is the optimal treatment protocol (27), since several rTMS variables significantly influence therapeutic efficacy, such as stimulation parameters and location. New ways to improve therapeutic effects should be further explored. For instance, new stimulation protocols such as theta burst stimulation (TBS) over the frontal cortex showed that intermittent TBS (iTBS, presumably causing facilitation similar to HF-rTMS) over the L-DLPFC and continuous TBS (cTBS, presumably resulting in suppression similar to LF-rTMS) over the R-DLPFC could improve the depressive symptoms (28, 29). Moreover, a new type of coil such as the H-coil to directly stimulate deeper brain regions have been developed and proven to be effective for the treatment of depression (30, 31). Further investigation should focus on improving the therapeutic effects rather than proving clinical efficacy.

Several directions may be suggested for the optimization of the clinical protocols. (1) Stimulation target. Most RCTs on depression to date have used the general “5-cm rule,” identifying the DLPFC target 5 cm anterior from the motor cortex site along the scalp surface corresponding to the abductor pollicis brevis muscle (32). However, several recent studies have shown that this location method is probably not optimal. Herbsman et al. found that more lateral and anterior stimulation over the prefrontal cortex provided a better antidepressant response (33), and Fitzgerald et al. showed that a neuronavigation method based on individual structural MRI was more effective than the standard 5 cm technique (34). A systematic comparison confirmed that DLPFC location based on individual structural MRI was ~2 cm anterior to that of the standard “5-cm rule” (35). Further simulation results based on electrical field and brain network showed that stimulation on the anterior and lateral DLPFC areas more likely to activate the executive network, while stimulation on posterior and medial DLPFC areas more likely to activate the default-mode network, causing different physiological consequences (36, 37). Fox et al. used a novel intrinsic (resting state fMRI) connectivity-based approach to gain insight into why some left DLPFC TMS targets have proven more clinically effective than the others, and they found that DLPFC TMS sites with better clinical efficacy were more negatively correlated with the activity of the subgenual cingulate (38). To summarize, further optimization of the DLPFC target via MRI-based anatomical location and/or EEG/fMRI-based functional location is needed (39).

(2) Dosage. As antidepressant medications, rTMS needs to accumulate “dosage” (affected by the intensity (%RMT), the pulse number per session and the session number) generate clinical efficacy. rTMS dosage in the RCTs on depression varied greatly, e.g., in intensity (80–120%), in the number of stimuli per session (120–3,000), and in the total number of treatment sessions (10–30) (2). The dosage is of importance, since it has been demonstrated that clinical efficacy for HF-rTMS over the L-DLPFC was higher for a higher number of sessions and rTMS pulses per session, and the rate of responders increased significantly when the total number of sessions was more than 10, the total number of pulses per session was more than 1,000, and the stimulation intensity was greater than 100% resting motor threshold (40). A recent meta-analysis found a similar influence of the stimulation parameters for LF-rTMS over the R-DLPFC, showing that more than 1200 pulses per session were needed to achieve high levels of response (12).

(3) Biomarkers. It remains unclear why some patients respond to rTMS treatment, while others do not; why some patients respond to HF-rTMS over the L-DLPFC, while others to LF-rTMS over the R-DLPFC, after more than 2 decades of exploration of rTMS treatment on primary depression. The reason is that the biomarkers of rTMS have not been elucidated. A very recent study showed that functional connectivity analysis based on resting-state fMRI could define depression subtypes and further predict responsiveness to TMS therapy (41). Additionally, TMS concurrent with EEG was of great use to study neural plasticity change and network reorganization induced by TMS therapy, highlighting the potential to elucidate TMS treatment-related biomarkers (42, 43).

### Limitations

First, only some of the studies (5/12) reported the remission rate in the present meta-analysis, and thus the remission rate analysis was less powerful than the response rate analysis. Second, we only examined the efficacy of TMS treatment immediately after each study's end, since very few studies reported long-term follow-up efficacy. Several studies showed that HF-rTMS efficacy could last for several months (44, 45). We could not estimate the stability of long-term antidepressant effects. This is important for future protocol optimization, since rTMS treatment sessions are labor-intensive and time-consuming for the patients (27). Third, we did not discriminate rTMS as a monotherapy or an augmentation strategy. Fourth, the conclusion was based on bipolar and unipolar patents grouped together, while ignoring the difference between the two populations. Fifth, we assessed both response and remission rates as treatment efficacy, as these two parameters are the two mostly used primary endpoints to evaluate depression treatment. We believe that the addition of continuous depression severity scores as an outcome in future studies will be more informative, and it probably improves rTMS treatment protocol. The last, in the current meta-analysis, we specifically focused on the efficacy comparison between the two most popular rTMS treatment protocols on depression: high frequency rTMS over left DLPFC vs. low frequency rTMS over right DLPFC. So, we only included the studies directly comparing the efficacy of the two protocols. We noticed that there is a latest meta-analysis on efficacy and acceptability of non-invasive brain stimulation (including different kinds of TMS and tDCS protocols) for the treatment of adult unipolar and bipolar depression (46), which is more comprehensive and informative.

## Author contributions

XC wrote the manuscript. CD and XS searched and analyzed data. YG polished the manuscript.

### Conflict of interest statement

The authors declare that the research was conducted in the absence of any commercial or financial relationships that could be construed as a potential conflict of interest.
